# Molecular Analysis, Pathogenic Mechanisms, and Readthrough Therapy on a Large Cohort of Kabuki Syndrome Patients

**DOI:** 10.1002/humu.22547

**Published:** 2014-03-13

**Authors:** Lucia Micale, Bartolomeo Augello, Claudia Maffeo, Angelo Selicorni, Federica Zucchetti, Carmela Fusco, Pasquelena De Nittis, Maria Teresa Pellico, Barbara Mandriani, Rita Fischetto, Loredana Boccone, Margherita Silengo, Elisa Biamino, Chiara Perria, Stefano Sotgiu, Gigliola Serra, Elisabetta Lapi, Marcella Neri, Alessandra Ferlini, Maria Luigia Cavaliere, Pietro Chiurazzi, Matteo Della Monica, Gioacchino Scarano, Francesca Faravelli, Paola Ferrari, Laura Mazzanti, Alba Pilotta, Maria Grazia Patricelli, Maria Francesca Bedeschi, Francesco Benedicenti, Paolo Prontera, Benedetta Toschi, Leonardo Salviati, Daniela Melis, Eliana Di Battista, Alessandra Vancini, Livia Garavelli, Leopoldo Zelante, Giuseppe Merla

**Affiliations:** 1 Medical Genetics Unit, IRCCS Casa Sollievo Della Sofferenza HospitalSan Giovanni Rotondo, Italy; 2 Ambulatorio Genetica Clinica Pediatrica, Clinica Pediatrica Università Milano Bicocca, Fondazione, MBBMAOS Gerardo Monza, Italy; 3 PhD Program, Molecular Genetics applied to Medical Sciences, University of BresciaBrescia, Italy; 4 U.O. Malattie Metaboliche Genetica Medica Endocrinologia; P.O. Giovanni XXIII, A.O.U. Policlinico ConsorzialeBari, Italy; 5 U.O. “Genetica Clinica e Malattie Rare” Ospedale Microcitemico CagliariItaly; 6 Department of Pediatrics, University of TurinTurin, 10126, Italy; 7 Section of Childhood and Adolescence Neuropsychiatry, Department Experimental and Clinical Medicine, University of SassariSassari, Italy; 8 Institute of Child Neuropsychiatry, University of SassariSassari, Italy; 9 Medical Genetics Unit, Children's Hospital Anna MeyerFirenze, Italy; 10 Department of Medical Science, Section of Medical Genetics, University of FerraraFerrara, Italy; 11 A.O.R.N.A. Cardarelli, U.O.S.C. Genetica MedicaNapoli, Italy; 12 Institute of Medical Genetics, Università Cattolica del Sacro Cuore, Largo Francesco Vito 1Rome, 00168, Italy; 13 UOC Genetica Medica, Azienda Ospedaliera RN “G.Rummo”Benevento, Italy; 14 Division of Medical Genetics, Galliera HospitalGenova, Italy; 15 Dipartimento Materno Infantile, Università degli Studi ModenaModena, Italy; 16 Rare Disease Unit, Paediatric Department, University of BolognaBologna, Italy; 17 Centro di Auxoendocrinologia, Department of Pediatrics, University of Brescia, Spedali CiviliBrescia, Italy; 18 Biologia Molecolare e Citogenetica, Diagnostica e Ricerca San RaffaeleMilano, Italy; 19 Medical Genetic Unit, Fondazione IRCCS Ca’ Granda Ospedale Maggiore, PoliclinicoMilan, Italy; 20 Department of Pediatrics, Genetic Counselling Service, Regional Hospital of BolzanoBolzano, Italy; 21 Medical Genetics Unit, University and Hospital of PerugiaPerugia, Italy; 22 U.O. Laboratorio di Genetica Medica, AOU PisanaPisa, Italy; 23 Clinical Genetics Unit, Department of Woman and Child Health, University of Padova, and IRP Città della SperanzaPadova, Italy; 24 Dipartimento di Pediatria, Area Funzionale di Genetica Clinica Pediatrica, Università degli Studi di Napoli “Federico II”Naples, Italy; 25 Clinica Pediatrica, IRCCS “G.Gaslini”, Università di GenovaGenova, Italy; 26 Newborn Intensive Care Unit, Maggiore HospitalBologna, Italy; 27 Clinical Genetics Unit, S.Maria Nuova Hospital, Reggio EmiliaItaly; 28 PhD Program, Scienze della Riproduzione e dello Sviluppo, University of TriesteTrieste, Italy

**Keywords:** KMT2D, KDM6A, Kabuki syndrome, haploinsufficiency, readthrough

## Abstract

Kabuki syndrome (KS) is a multiple congenital anomalies syndrome characterized by characteristic facial features and varying degrees of mental retardation, caused by mutations in *KMT2D*/*MLL2* and *KDM6A*/*UTX* genes. In this study, we performed a mutational screening on 303 Kabuki patients by direct sequencing, MLPA, and quantitative PCR identifying 133 *KMT2D*, 62 never described before, and four *KDM6A* mutations, three of them are novel. We found that a number of *KMT2D* truncating mutations result in mRNA degradation through the nonsense-mediated mRNA decay, contributing to protein haploinsufficiency. Furthermore, we demonstrated that the reduction of KMT2D protein level in patients’ lymphoblastoid and skin fibroblast cell lines carrying *KMT2D*-truncating mutations affects the expression levels of known *KMT2D* target genes. Finally, we hypothesized that the KS patients may benefit from a readthrough therapy to restore physiological levels of KMT2D and KDM6A proteins. To assess this, we performed a proof-of-principle study on 14 *KMT2D* and two *KDM6A* nonsense mutations using specific compounds that mediate translational readthrough and thereby stimulate the re-expression of full-length functional proteins. Our experimental data showed that both *KMT2D* and *KDM6A* nonsense mutations displayed high levels of readthrough in response to gentamicin treatment, paving the way to further studies aimed at eventually treating some Kabuki patients with readthrough inducers.

## Introduction

Histone methylation is an epigenetic mechanism by which spatial and temporal expression of distinct genes and pathways are regulated at precise developmental stages. Different histone lysine methylation states (mono-, di-, or tri-methylation) are associated with gene transcriptional activation or repression depending on the location of the lysine residue.

In general, histone H3 lysine 4 (H3K4) di- and tri-methylation are linked to active transcription, whereas H3K27 di- and tri-methylation are associated with gene silencing [Santos-Rosa et al., 2002]. Aberrations in the histone modifiers have been associated with genetic diseases, such as Kleefstra syndrome, Sotos syndrome, Weaver syndrome, and Schinzel–Giedion syndrome [Berdasco and Esteller, 2013]. The discovery of histone methyltransferase (HMT) KMT2D (MIM #602113; RefSeq NM_003482.3, also known as MLL2, ALR/MLL4) and demethylase KDM6A (MIM #300128; RefSeq NM_021140.2, also known as *UTX*) genetic alterations in Kabuki syndrome (KS) patients expanded and highlighted the role of histone modifiers in causing congenital anomalies and intellectual disability [Bogershausen and Wollnik, 2013]. KS (MIM #147920) is an autosomal-dominant condition characterized by striking facial features, such as elongated palpebral fissures with eversion of the lateral third of the lower eyelid, short columella with depressed nasal tip, skeletal anomalies, dermatoglyphic abnormalities, mild-to-moderate mental retardation, and postnatal growth deficiency [Kuroki et al., 1981; Niikawa et al., 1981]. KS is commonly associated with congenital heart defects, genitourinary anomalies, cleft lip and/or palate, susceptibility to infections, gastrointestinal abnormalities, ophthalmologic defects, ptosis and strabismus, dental anomalies, including widely spaced teeth and hypodontia, and ear pits. Additionally, KS individuals might have a number of less frequent findings comprising visceral abnormalities and premature breast development in females.

In 2010, whole-exome sequencing successfully identified heterozygous mutations in the *KMT2D* gene as the major cause of KS [Ng et al., 2010]. Since then, 55%–65% of KS cases have been reported carrying a *KMT2D* mutation [Ng et al., 2010; Hannibal et al., 2011; Li et al., 2011; Micale et al., 2011; Paulussen et al., 2011; Banka et al., 2012, 2013; Makrythanasis et al., 2013; Miyake et al., 2013a]. The majority of mutations identified were de novo, although familial cases with autosomal-dominant inheritance have occasionally been described [Hannibal et al., 2011; Kokitsu-Nakata et al., 2012]. *KMT2D* gene maps to 12q13.12 and encodes a gigantic protein (5,537 residues) that belongs to the mixed lineage leukemia (MLL) family of HMTs. The MLL proteins are part of the SET (Su[var]3–9, enhancer-of-zeste, Trithorax) family of proteins [Dillon et al., 2005] that play important roles in the epigenetic control of active chromatin states [Issaeva et al., 2007]. They act as transcriptional coactivators and are involved in the expression control of genes essential for embryogenesis and development such as the *HOX* genes [Ansari and Mandal, 2010; Eissenberg and Shilatifard, 2010].

A subset of KS individuals was recently identified with either point mutations or microdeletions encompassing the X-linked gene, *KDM6A* [Lederer et al., 2012; Miyake et al., 2013a; Miyake et al., 2013b] that encodes a Histone H3 lysine-27 demethylase. KDM6A plays a crucial role in general chromatin remodeling [Hong et al., 2007; Lan et al., 2007] and interacts with KMT2D, in a conserved SET-1-like complex that trimethylates H3K4 [Issaeva et al., 2007]. The inactivation of the zebrafish *kdm6a* orthologue by morpholino is associated with severe and diverse structural defects and developmental abnormalities [Lindgren et al., 2013]; this inactivation resulted in the misregulation of *HOX* genes leading to a posterior developmental defect, whereas *Kdm6a*-deficient mice showed severe defects in heart development and embryonic lethality [Lee et al., 2012].

The majority of *KMT2D* and *KDM6A* nucleotide changes are truncating mutations (nonsense, frameshift, or splice site) that produce premature termination codons (PTCs), which are potentially deleterious. In the last few years, there have been several attempts to develop mutation-specific pharmacological approaches to restore sufficient levels of functional proteins. One approach that has been gaining prominence is that of using pharmacological agents to promote nonsense suppression or readthrough of PTCs thus enabling re-expression of full-length functional proteins [Nakamura et al., 2005; Bellais et al., 2010]. The potential of aminoglycosides and nonaminoglycosides as therapeutic tools has been demonstrated in several genetic disorders such as hemophilia, β-thalassemia, and spinal muscular atrophy, but most extensively in Duchenne muscular dystrophy and cystic fibrosis [Lee and Dougherty, 2012]. Interestingly, this treatment was successfully applied on an Ataxia-telangiectasia patient with heterozygous nonsense mutation, thereby demonstrating therapeutic ability despite the presence of a nonsense mutation in just one allele [Nakamura et al., 2011].

In this report, we have expanded the spectrum of mutations of *KMT2D* and *KDM6A* genes by analyzing our cohort of 303 Kabuki patients by direct sequencing, MLPA and quantitative PCR (qPCR). Based on KMT2D biological role, we designed functional studies that highlighted the haploinsufficiency of KMT2D as one of the mechanisms underlying the pathogenesis of the disease. Moreover, we evaluated the readthrough efficiency of 14 *KMT2D* and two *KDM6A* nonsense mutations and showed that 11 *KMT2D* and one *KDM6A* nonsense mutation responded to gentamicin treatment suggesting that this strategy can be effective to restore functional endogenous protein level and biological activity of KMT2D and KDM6A.

## Material and Methods

### Patients

In this study, 303 KS patients were included following the inclusions criteria reported in Micale et al. (2011). Patients were enrolled after obtaining appropriate informed consent by the physicians in charge and approval by the respective local ethics committees.

### Cell Cultures, Nonsense-Mediated mRNA Decay Assay, and E2 Treatment

Lymphoblastoid cell lines (LCLs) were established from fresh peripheral blood leukocytes, infected by Epstein Barr Virus and cultured in RPMI 1640 supplemented with 10% of fetal bovine serum (FBS; Life Technologies, USA), l-glutamine, and 1% antibiotics mixture (penicillin and streptomycin 10,000 UI/ml; Life Technologies). Primary skin fibroblasts were grown in minimum essential medium supplemented with 1% l-glutamine, 10% FBS, and antibiotics. Nonsense-mediated mRNA decay (NMD) was assayed by treating fibroblast and lymphoblast cell lines with puromycin at a concentration of 200 μg/ml. After 8 hr of incubation, total RNA was obtained using the RNasy mini Kit (Qiagen, Germany) according to manufacturer instructions and Quantitect Reverse Transcription kit (Qiagen, Düsseldorf, Germany) was used for cDNA synthesis. Fibroblast cell lines were treated with 200 nM of 17B-estradiol (E2), incubated for 8 hr and then harvested for RNA and protein extraction.

### MLPA, Long PCR, and Genomic Real-Time qPCR

Genomic DNAs were extracted from fresh and/or frozen peripheral blood leukocytes of the probands and their parents following standard procedures. MLPA analysis was performed as reported in Priolo et al. (2012) using probe mixture (SALSA MLPA KIT P389-A1 *KMT2D*; MRC-Holland, Amsterdam, The Netherlands) that contains 27 probes targeting exons across the *KMT2D* gene.

Long PCR was carried out with Expand Long Template PCR system (Roche, Mannheim, Germany) with combinations of primer pairs spanning the *KMT2D* exons not covered by MLPA. qPCR reactions were carried out with primers designed to amplify all the 29 *KDM6A* exons as described in Priolo et al. (2012).

### Real-Time qPCR Assays

Total RNA was extracted from peripheral blood leukocytes using TRIZOL reagent (Life Technology) and reverse transcribed using the Quantitect Transcription kit (Qiagen), according to the manufacturer's instructions. Oligos for qPCR were designed using the Primer express program [Rozen and Skaletsky, 2000] with default parameters. *EEF1A1* and *GAPDH* were used as references genes. qPCR reactions and calculations were made as reported in Ferrero et al. (2010). Significance was determined by a two-tailed unpaired *t*-test for means.

### Dual Luciferase Reporter Vector System

A dual gene reporter pCRFL (gently provided by Prof. J-P Rousset) was used to quantify the effect of gentamicin on stop mutation readthrough in culture cells. Sequence to be analyzed spanning 27 nucleotides centered on the different stop mutations were inserted in-frame between the *Renilla* and *Firefly* Luciferase coding sequences through site-directed mutagenesis by using the QuickChange II kit (Stratagene, La Jolla, California, USA). All target DNA sequences were assessed by sequencing. Plasmids were purified using Qiagen Midiprep Kit according to the manufacturer's specifications. HEK293 cells were culture in Dulbecco's modified Eagle's medium supplemented with 10% FBS, 1% antibiotics mixture, and incubated at 37% in humidity saturated 5% CO_2_. Cells were transfected using FuGene HD Transfection Reagent (Promega) with each pCRFL construct. Culture medium was replaced 24 hr later with fresh medium at a concentration of gentamicin (Hospira, Lake Forest, Illinois, USA) varying from 0 to 1,200 μg/ml. After 48 hr, the cells were lysed in passive lysis buffer and assayed for both *Firefly* and *Renilla* luciferase activity using the Dual-GLO® Luciferase Assay System (Promega, Madison, WI, USA).

This dual reporter allows the quantification of stop-codon readthrough, by measuring *Firefly* and *Renilla* activities, as previously described [Bidou et al., 2004].

Briefly, readthrough efficiency was estimated by comparing the *Firelfly*/*Renilla* luciferase ratio obtained for each nonsense mutation to an in-frame control. A 100% activity control was provided by a construct (TQ) with no stop codon between the coding sequences of the two reporters [Sermet-Gaudelus et al., 2007]. A pCRFL reporter vector harboring the 319d Duchenne muscular dystrophy mutation (pCRFL319), exhibiting the highest gentamicin-induced readthrough efficiency and the highest induction factor in NIH3T3-cultured cells assays [Bidou et al., 2004], was used as positive control. Values are the mean ± SEM of three experimental replicates from three independent transfections. Significance was determined by a two-tailed unpaired *t*-test for means.

### PCR-Based Sequencing of *KMT2D* and *KDM6A*

Mutation screening of all 54 coding exons of the *KMT2D* gene was performed as reported in Micale et al. (2011). *KDM6A* (NM_021140.2) primers were designed to amplify exons and adjacent splice sites according to the reference sequences, using the Primer 3 Output program (http://frodo.wi.mit.edu/primer3/). A complete list of primer sequences and PCR conditions are available on request. The amplified products were subsequently purified and sequenced with a ready reaction kit (BigDye Terminator v1.1 Cycle; Warrington WA1 4SR, UK). The fragments obtained were purified using DyeEx plates (Qiagen) and resolved on an automated sequencer (3130xl Genetyc analyzer DNA Analyzer; ABI Prism). Sequences were analyzed using the Sequencer software (Gene Codes, Ann Arbor, MI). We resequenced all identified mutations in independent experiments. The following databases were used to obtain gene information: National Center for Biotechnology Information (NCBI, http://www.ncbi.nlm.nih.gov/), Ensembl Genome Server (http://www.ensembl.org/), UCSC Genome Bioinformatics (http://www.genome.ucsc.edu/), and 1000 genomes (http://browser.1000genomes.org/). All existing and new mutations were described following the recommendations of the Human Genome Variation Society (http://www.hgvs.org/mutnomen).

### In Silico Analysis

We analyzed the missense variants by the latest version of the server Polyphen-2 version 2.2.2 (http://genetics.bwh.harvard.edu/pph) (Adzhubei et al., 2010), Align GVGD (http://agvgd.iarc.fr/agvgd_input.php) [Tavtigian et al., 2006], PROVEAN v1.1 (http://provean.jcvi.org/index.php) [Choi et al., 2012], and SIFT v1.03 (http://sift.jcvi.org/) [Kumar et al., 2009], UMD-predictor (http://www.umd.be/) [Frederic et al., 2009], using default parameters. Splice-site variants were evaluated for putative alteration of regulatory process at the transcriptional or splicing level with NetGene2 (http://www.cbs.dtu.dk/services/NetGene2) [Brunak et al., 1991] and NNSPLICE (http://www.fruitfly.org/seq_tools/splice.html) [Reese et al., 1997].

### Protein Extraction and Western Blot Analysis

Normal and patient lymphoblastoid and fibroblast cell lines were treated with concentration of gentamicin varying from 800 to 1,200 μg/ml. After 48 hr, cells were lysed in 10-mM HEPES, 1.5-mM MgCl2, 10-mM KCl, 0.5-mM DTT, 1.5-mM Phenylmethylsulfonyl fluoride (PMSF), and 2 N KCl. Proteins were separated on a SDS-polyacrylamide gel. Western blots were prepared as reported in Caratozzolo et al. (2012) and probed with anti-KMT2D (Abnova, Taipei, Taiwan). Bound primary antibodies were visualized using ECL western blotting or ECL plus Western blotting detection reagents (GE Healthcare, UK).

## Results

### Mutation Screening of *KMT2D* and *KDM6A*

Since 2011, we performed a comprehensive mutational screening on 303 Kabuki patients by direct sequencing, MLPA, and qPCR on *KMT2D* and *KDM6A* genes, respectively. Of these 303 patients, 79 were described in previous studies where 53 pathogenic *KMT2D* mutations were identified in 51 patients (Supp. Table S1) [Micale et al., 2011; Makrythanasis et al., 2013; Ratbi et al., 2013].

Here, we extended the *KMT2D* mutational analysis to a new cohort of 224 individuals clinically diagnosed as KS identifying 82 (82/224, 36%) patients carrying causative *KMT2D* mutations.

Overall, in our whole KS cohort, we identified 133/303 (34%) patients with *KMT2D* mutations and 140 different *KMT2D* mutations; of them, 87/140 (62%) were identified in this study with 66/140 (47%) never described before. The following types of *KMT2D* mutations were identified: 37 nonsense (37/140, 26%), 42 frameshift (42/140, 30%), 46 missense (46/140, 33%), eight splice site (8/140, 6%), and seven indel (7/140, 5%) (Supp. Tables S1 and S2). Almost all identified mutations occurred de novo; only 16 missense and four indel variants were inherited from an apparently asymptomatic parent.

To explore the occurrence of intragenic deletions and duplications, we screened *KMT2D* exons by MLPA analysis on 207 KS patients: 164 *KMT2D* point mutation negative, 37 with *KMT2D* missense, and six indels. We identified the KB43 patient carrying a singleton deletion covering from exon 49 to a part of exon 51 (Supp. Fig. S1A). Long PCR, followed by direct sequencing, allowed us to map the boundaries of the deletion as c.15785-238_16168del2425insTTGTATCTCAA mutation (Supp. Fig. S1B). KMT2D mutations were submitted to the Leiden Open Variation Database (LOVD v.3.0; http://www.lovd.nl/3.0/home) public database.

To identify *KDM6A* mutations in KS patients, we first assessed *KDM6A* exon dosage by qPCR analysis on 139 samples with no *KMT2D* alteration without identifying any intragenic deletions and duplications. Then, we sequenced 29 coding exons of *KDM6A* along with its exon–intron boundaries in 98 patients (not enough DNA was available for the remaining 41 samples). We identified four *KDM6A* point mutations: one nonsense c.514C>T (p.Arg172X) [Banka et al., 2014], one frameshift c.1846_1849delACTC (p.Thr616TyrfsX8), one missense c.2939A>T (p.Asp980Val), and one splicing mutation c.3284+3_3284+6delAAGT (p.Asn1070_Lys1094del). Two of them occurred de novo; parental DNAs were unavailable for the others. *KDM6A* mutations were submitted to LOVD (http://www.lovd.nl/3.0/home) public database.

### Characterization of *KMT2D* Splice-Site Mutations

We identified eight patients with *KMT2D* mutations in the proximity of the splice sites and we analyzed them by NetGene2 and Fruitfly softwares to predict the molecular consequences of the observed nucleotide changes. The availability of the RNA from those KS patients, but one, allowed us to experimentally determine the effect at the transcriptional or splicing level (Supp. Fig. S2). All identified intronic *KMT2D* variants cause aberrant splicing of the corresponding transcripts that result in a frameshift with the generation of a premature stop codon as reported below:

- c.177-2A>C (p.Ser59ArgfsX86) affects the essential nucleotide −2 of the splice acceptor site of intron 2. Real-time PCR analysis and fragment sequencing of the carrier patient revealed skipping of the entire exon 3 (Supp. Fig. S2A).
- c.400+1G>A (p.Ser59ArgfsX86) occurring within the GT splice donor site in intron 3 resulted in the disruption of the canonical splice site and again in skipping of exon 3 (Supp. Fig. S2B).
- c.400-3A>G (p.Gly134GlufsX74) creates a novel AG splice acceptor site in intron 3. Sequencing of the normal-sized fragment amplified from patient's cDNA revealed a transcript with an insertion of an AG dinucleotide that corresponds to the effective splice-site acceptor of intron 3 (Supp. Fig. S2C).
- c.13999+5G>A (p.Asn4614IlefsX5) occurs at nucleotide +5 of the splice donor site of intron 42. Sequencing analysis of the real-time PCR fragments confirmed that the observed shorter transcript results from a splicing failure and exclusion of exon 42 in the mRNA (Supp. Fig. S2D).
- c.14252-6_14252-5insGAAA (p.Val4751_GlufsX22) consists of a four nucleotides insertion in intron 44 that causes skipping of the entire exon 45 (Supp. Fig. S2E).
- c.14643+1G>A (p.Gln4882ProfsX36) occurring at nucleotide +1 of the splice donor site of intron 47 creates a novel splice acceptor site within exon 48. The PCR products from patient's cDNA showed two bands, one of the expected size, and an additional shorter band carrying a partial deletion of the exon 48 (Supp. Fig. S2F).
- c.14644-3C>G (p.Glu4882ProfsX36) occurring at nucleotide −3 of the splice acceptor site of intron 47 determine the same molecular events caused by c.14643+1G>A mutation (Supp. Fig. S2G).
- c.4693+1G>A (p.Val1561ArgfsX11) mutation was previously characterized and reported by Ratbi et al. (2013).

### *KMT2D* Mutant Transcripts Suffer NMD

*KMT2D* nonsense mutations may result in the partial transcripts degradation through NMD pathway, contributing to protein haploinsufficiency. To investigate this, we measured by qPCR the levels of *KMT2D* mRNA in three KS skin fibroblasts and four KS LCLs following treatment with puromycin, a known indirect NMD inhibitor [Brichta et al., 2008]. After puromycin treatment, we observed a significant 2.18-, 2.35-, and 4.01-fold increase in the *KMT2D* transcript levels in KB186, KB153, and KB3 fibroblast cell lines, respectively (Supp. Fig. S3A and Supp. Table S3E), and 1.93- and 1.69-fold for KB48 and KB83 LCLs, compared with untreated cells, respectively (Supp. Fig. S3C and Supp. Table S3F). The reduced amount of *KMT2D* mRNA levels in mutated cell lines and its recovery following puromycin treatment indicate that endogenous *KMT2D* mutant transcripts are subject to NMD. The physiological NMD substrate SC-35 1.7 Kb was included as positive control (Supp. Fig. S3B–D) [Brichta et al., 2008]. We confirmed these data by direct sequencing of real-time PCR on mRNA derived from fibroblasts of patients KB186, KB153, KB3, showing that NMD inhibition restores the expression of the *KMT2D*-mutated alleles (Supp. Fig. S3E–G).

### *KMT2D* Mutations Affect Its Activity in Patients Cell Lines

**Figure 1:**
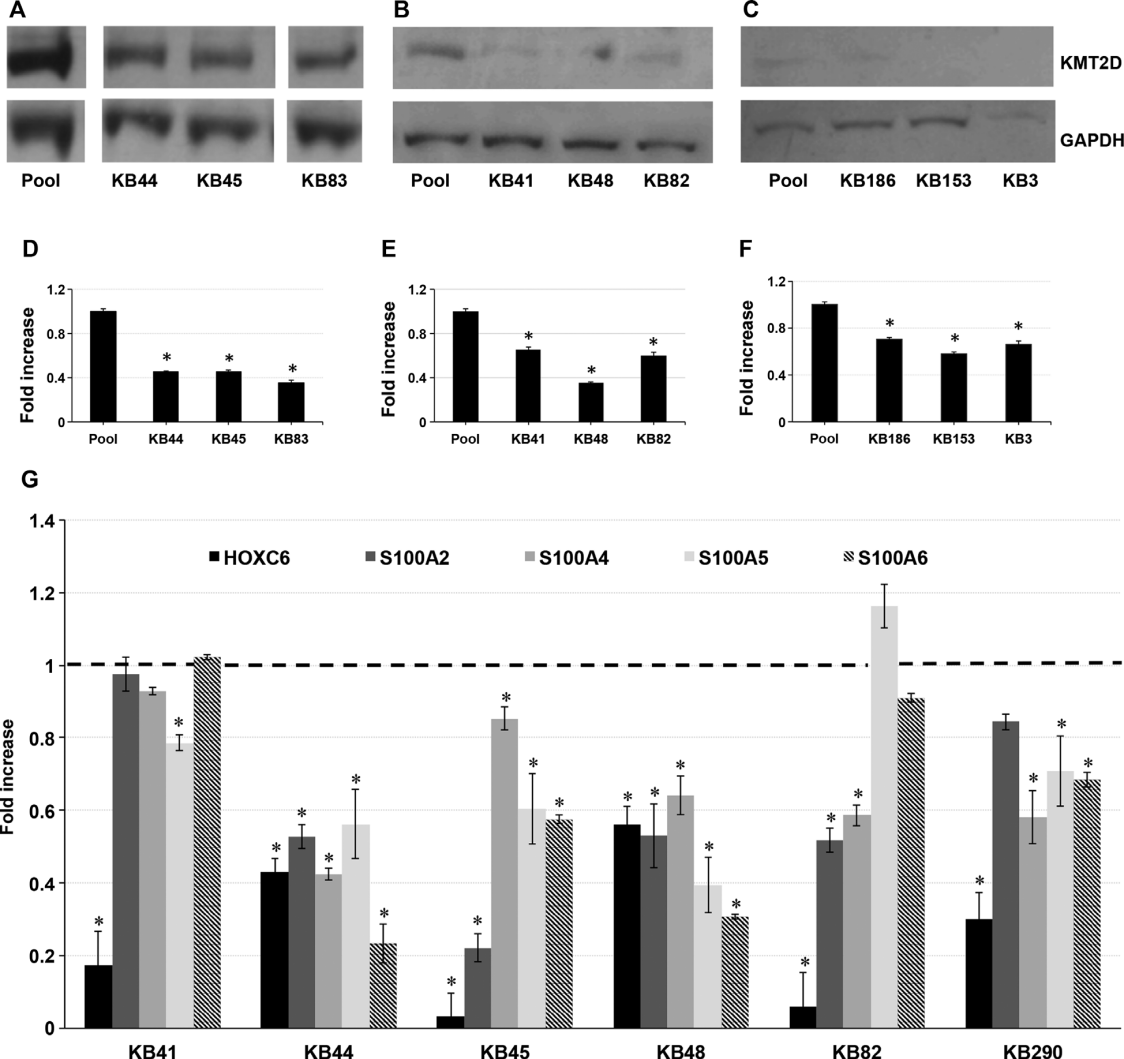
KMT2D methyltransferase activity is impaired in KS lymphoblastoid and fibroblast cell lines with KMT2D-truncating mutations. (A–F): Immunoblotting analysis by using anti-KMT2D antibody on whole protein lysate from six (A and B) KS lymphoblastoid (KB41, KB44, KB45, and KB82, KB83, KB153, and KB186) and three (C) fibroblast cell lines (KB186, KB153, and KB3) with nonsense mutations and frameshift mutations, compared with control cell lines. Pool: pooled protein lysates from two control cell lines. D–F: The density of each band was determined by densitometer. The expression level of KMT2D was determined by calculating the protein level for each sample, normalized to the corresponding GAPDH level. G: qPCR was performed to measure the expression of known target genes of *KMT2D* (*HOXC6, S100A2, S100A4, S100A5, S100A6*) in six KS–LCLs, compared with control cell lines. Scale bars represent standard errors. *P < 0.01.

The large prevalence of *KMT2D* protein truncating mutations suggests loss of function, and therefore the haploinsufficiency of KMT2D as the likely mechanism for the KS phenotype. To assess this, we measured the protein level of KMT2D in six KS LCLs, and three KS fibroblast cell lines carrying truncating mutations, and we found a significant reduction of KMT2D protein level when compared with control cell lines (Fig.1A–F and Supp. Table S3A).

To address whether truncated *KMT2D* mutations affect its transcriptional activity, we measured the mRNA level of direct known transcriptional target genes of KMT2D complex, including *HOXC6* [Ansari et al., 2011] and some members of the *S100A* family genes, such as *S100A2*, *S100A4*, *S100A5*, and *S100A6*. As shown in Figure1G, KMT2D targets were downregulated in KS LCLs cells (Supp. Table S3B). Overall, these data show that the transcriptional activity of KMT2D is impaired in KS patients.

### *HOXC6* Expression Is Affected in Kabuki Patients

**Figure 2:**
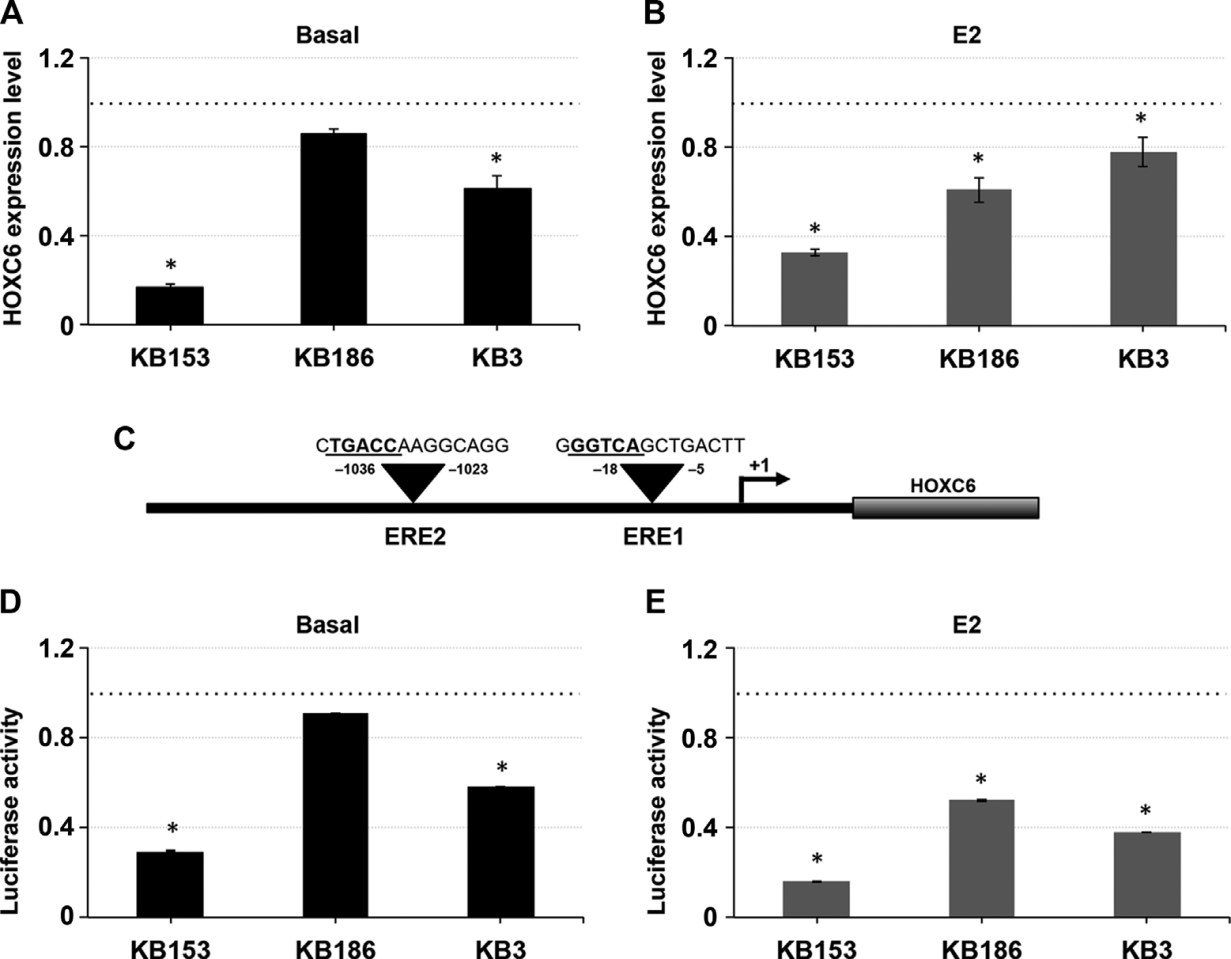
Effect of KMT2D haploinsufficiency on E2-induced expression of *HOXC6*. A and B: *HOXC6* transcriptional level was determined in three KS patient fibroblast cell lines (KB153, KB186, and KB3) exposed to 100 nM E2 for 8 hr or not by using qPCR analysis, in comparison to a pool of two normal fibroblast cell lines. C–E: *HOXC6* promoter fragment spanning the ERE1–ERE2 regions was cloned into a luciferase-based reporter construct (pGL3) and transfected into three KS patient fibroblast cell lines and into two normal fibroblast cell lines along with a Renilla luciferase construct used as an internal transfection control. Cells were then treated with 100 nM E2 (E) for 8 hr and subjected to luciferase assay. The luciferase activities (normalized to Renilla activity) in the presence or not of E2 over normal fibroblast cell lines were plotted. The experiment with three replicates was repeated at least thrice. Scale bars represent standard errors. *P < 0.01.

Many of the *KMT2D* mutations mapped before the LXXLL domain required for KMT2D binding to estrogen receptors (ERs), ERα, and ERβ. Previous studies demonstrated that KMT2D, in coordination with ERα and ERβ, transcriptionally regulates *HOXC6* in an Estradiol (E2)-dependent manner. We thus hypothesized that the loss of the KMT2D–ER interaction affects ER gene expression. qPCR showed that *HOXC6* transcriptional level decreased in KS fibroblast cells, a reduction that persists even after E2 treatment (Fig.2A and B and Supp. Table S3C). As KMT2D is associated with *HOXC6* via binding to the estrogen receptor element (ERE), located in the *HOXC6* promoter region, we assessed the ability of KMT2D to bind to the *HOXC6* EREs in three KS patients by a luciferase reporter assay. We transfected the pGL3–ERE1–ERE2 luciferase-based reporter vector containing the *HOXC6* EREs sequences (schematized in Fig.2C) in KS and healthy fibroblast cell lines treated with E2 for 8 hr, then we measured the luciferase activity. Using a two-tailed unpaired *t*-test, we detected a significant reduction of the luciferase activity in all patient fibroblast cell lines (Fig.2D and E and Supp. Table S3D), in both treated or not with E2, when compared with control cells transfected with pGL3 alone. Overall, these data indicate that *KMT2D* mutations impair the regulation of *HOXC6* in Kabuki cell lines.

### KMT2D and KDM6A Nonsense Mutations Are Responsive to Gentamicin-Induced Readthrough Treatment

**Figure 3:**
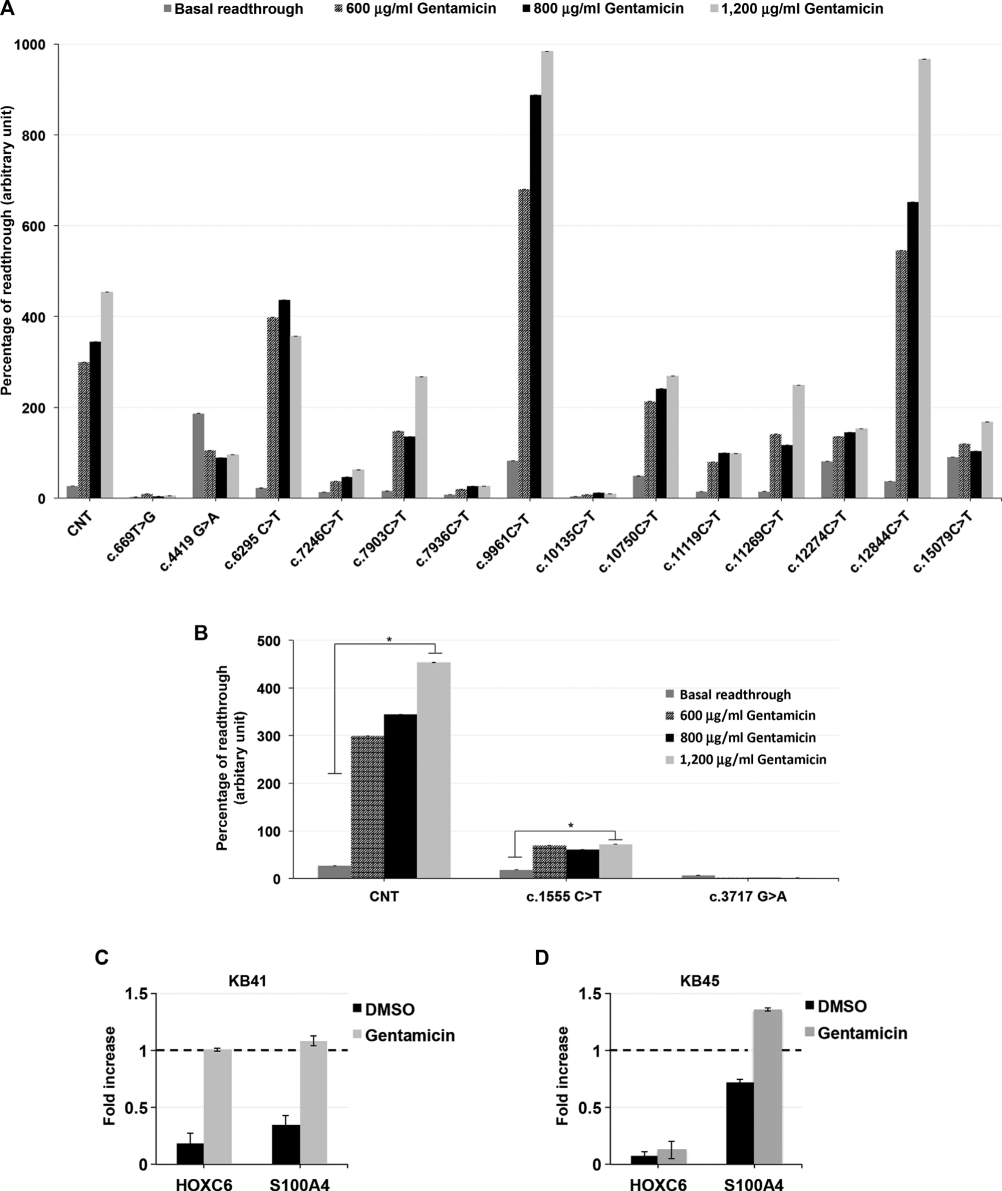
Identification of *KMT2D* and *KDM6A* nonsense mutations responsive to gentamicin treatment. A and B: Comparison between basal and gentamicin-induced readthrough level. Readthrough efficiency for 14 nonsense mutations in the *KMT2D* gene and for two elsewhere published [Miyake et al., 2013b] nonsense mutations in the *KDM6A* gene was assessed in HEK293 cells exposed or not to 600–800–1,200 μg/ml of gentamicin for 48 hr. A pCRFL reporter vector harboring the 319d Duchenne muscular dystrophy mutation was used as positive control (CNT). Each value corresponds to the mean of four to six independent experiments. Scale bars represent standard errors. C and D: Gentamicin restored the expression of *HOXC6* and *S100A4* in patient cultured cells with different efficiency. qPCR analysis was performed to evaluated *HOXC6* and *S100A4* expression level in two patients LCLs, KB41 (C) and KB45 (D), and two control cell lines treated or not with 800 μg/ml of gentamicin for 48 hr. The results are the mean from at least two experiments performed in duplicate. *P < 0.01.

A large number of *KMT2D* and *KDM6A* mutations are nonsense mutations that produce aberrant PTCs that are potentially deleterious. We evaluated the potential ability of gentamicin in suppressing *KMT2D* and *KDM6A* nonsense mutations by using an in vitro readthrough reporter system [Floquet et al., 2011]. Fourteen *KMT2D* and two *KDM6A* nonsense mutations were selected and tested. The mutations were selected, when possible, following the hypothetical rule of a uracil immediately upstream and a cytosine just downstream the stop codon as associated with an optimal gentamicin-induced readthrough, respectively [Floquet et al., 2012]. For each mutation, sequences spanning 27 nucleotides centered on the different stop mutations were inserted into the dual reporter vector pCRFL in-frame between Renilla and Firefly luciferase coding sequences [Floquet et al., 2011]. Readthrough levels were quantified in HEK293 cells transiently transfected by the dual reporter vector in the presence of increasing amounts of gentamicin. Cytotoxicity experiments demonstrated that no signs of cytotoxicity were detectable after 48 hr of treatment with up to 1,200 μg/ml of gentamicin (data not shown). The difference between basal and gentamicin-induced readthrough level was statistically significant (*P* < 0.01) for 11 *KMT2D and one KDM6A* nonsense mutations (Fig.3A and B and Supp. Table S4), although with a wide variability in strength. In agreement with the previous report [Floquet et al., 2012], we found that 11/12 (92%) of the mutations responsive to gentamicin have a uracil in −1 residue immediately upstream the stop codon and/or a cytosine in +4 position confirming that the presence of these residues promotes higher basal and gentamicin-induced readthrough than other nucleotides. To test the in vivo effectiveness of gentamicin, we evaluated the expression levels of two known targets of KMT2D, *HOXC6*, and *S100A4* in LCLs from KS patients cultured in the presence of gentamicin. qPCR analysis revealed that KB41 LCL resulted in sixfold and threefold increase of *HOXC6* and *S100A4* mRNA levels, respectively (Fig.3C). In agreement with the in vitro assay, a significant increase of *S100A4* mRNA levels was observed in KB45 cells, whereas we did not detect any significant increase of *HOXC6* expression (Fig.3D). These findings are consistent with our in vitro observations about the effectiveness of readthrough that varies with respect to type of mutations and patient cell lines.

Overall, these studies suggest that readthrough strategies can be effective to restore functional KMT2D and KDM6A activity, although testing additional mutations, many other compounds and

different cell lines are mandatory to assess their potential therapeutic effectiveness.

## Discussion

The orchestrated organization of epigenetic factors that control chromatin dynamism, including chromatin-remodeling proteins, is essential for the proper function of tissue homeostasis, cell identity, and development [Berdasco and Esteller, 2013]. KS is the most common of a growing group of multiple malformation syndromes associated to developmental delay that are caused by mutations in genes that encode proteins involved in histone methylation. In this study, we identified 133 *KMT2D* and four *KDM6A* mutations in 303 patients clinically diagnosed with KS. The mutation detection rates for *KMT2D* and *KDM6A* in KS suggest that other causative genes may be involved, although it can be taken into account that a number of these patients might have been misdiagnosed.

We demonstrated that KMT2D protein level is reduced and its activity impaired in KS cell lines. KMT2D through H3K4 methylation activity is a key epigenetic transcriptional regulator of the expression of multiple genes with related functions, including embryogenesis, development, and stem cell differentiation. Additionally, KMT2D-associated histone methyl transferase activity appears to be functional only in the context of its multiprotein complex, including ASH2L, RBBP5, WDR5, and other MLL family members [Issaeva et al., 2007; Dhar et al., 2012] and each KMT2D-interacting protein plays a distinct role in regulating MLL-mediated histone methylation and gene activation. In agreement with its biological function, the lack of KMT2D protein activity or of its ability to form a multiprotein complex might alter its role in histone methylation pathway with strong influences on global change in gene expression in many of the body's organs and tissues, resulting in some of the abnormalities of postnatal development of KS.

In our screening, we found 46 missense variants distributed across the entire length of the *KMT2D* gene including residues in PHD and SET domain. The in silico analysis predicts that many of them might be pathogenic, although the exact role they play in the disease has not been addressed. Recently, it has been shown that KS C1430R and C1471Y missense mutations [Hannibal et al., 2011] in PHD_4–6_ domains reduce PHD_4–6_ binding ability and abrogate the nucleosomal methylation activity of *KMT2D*, although they do not affect the interaction of KMT2D with ASH2L, RBBP5, and WDR5 [Dhar et al., 2012]. These results offer a valuable and feasible assay to test the pathogenicity of *KMT2D* missense mutations and implicate that the missense mutations described here that lies in PHD domains may contribute to KS by reducing KMT2D enzymatic activity and consequently its mediated transcriptional activation.

The majority of genetic alterations detected in Kabuki patients result in truncated proteins upstream the LXXLL domain, which is involved in KMT2D-complex binding to ERα. Rationally, the loss of the KMT2D–ERα interacting region might result in a dramatic alteration of the ERα-mediated pathways. Notably, some of the clinical features associated to deregulated ERα-signaling pathways are included in the large spectrum of KS phenotypes, comprising immunological defects and cardiac anomalies [Deroo and Korach, 2006]. Moreover, more than 20% of Kabuki patients show early breast development consistent with the biological role of ERα in mammary gland formation. In this regard, it was recently reported that the homeobox (HOX)-containing gene *HOXC6*, a critical player in mammary gland development and milk production, is transcriptionally activated via coordination of KMT2D and ER in an estrogen environment in breast cancer and placental choriocarcinoma cells. Specifically, the HMTs, KMT2D, and MLL3, in coordination with ERs, ERα, and ERβ, play critical roles in histone H3 lysine-4 trimethylation and in the recruitment of general transcription factors and RNA polymerase II in the EREs regions of the *HOXC6* promoter during E2-dependent transactivation, leading to *HOXC6* transcriptional activation. Interestingly, the knockdown of *KMT2D* by using specific antisense oligonucleotides suppressed E2-induced expression of *HOXC6* [Ansari et al., 2011]. In agreement with all these findings, we found that *HOXC6* expression is impaired in KS patients’ cell lines.

As Kabuki patients’ cell lines with *KDM6A* mutations were not available, it was not possible to study the activity of the endogenous KDM6A. However, it has been recently evaluated that the haploinsufficiency of *Kdm6a* in a zebrafish model. *Kdm6a* knockdown fish exhibited abnormal craniofacial structures that included the absence of the branchial arches and otoliths, as well as the absence, clefting, or inversion of the ceratohyal and abnormal patterning or clefting of Meckel's cartilages [Lindgren et al., 2013]. In addition, Kdm6a-deficient mice showed severe defects in heart development and embryonic lethality [Lee et al., 2012]. Very recently, it was demonstrated that *Kdm6a* knockdown affects expression of master regulatory genes involved in adipogenesis and osteogenesis [Hemming et al., 2014]. These observations further support the hypothesis that perturbation of a regulatory pathway shared by KDM6A is responsible for the clinical aspects of the KS.

The large size of the *KMT2D* cDNA hampers so far the development of any gene-therapy-based strategy. Mutation-based treatments are something of recent enough in genetic medicine, in which the nature of the mutation dictates the therapeutic strategy. As proof of principle, here we demonstrated the ability of gentamicin to induce the readthrough of naturally occurring stop mutations in the *KMT2D* and *KDM6A* genes using both in vitro and in vivo assays. Readthrough efficiency has been shown to also depend on the nature of the sequences surrounding the stop codon. Specifically, the consensus sequence U-STOP-C results in an optimal gentamicin-induced readthrough [Floquet et al., 2012]. However, the readthrough response to treatment is highly variable and little is known about the rules governing it and the response to different compounds. A wide variability in responsiveness to gentamicin was observed in our group of *KMT2D* and *KDM6A* mutations. Interestingly, the c.12844C>T mutation that exhibited the highest gentamicin-induced readthrough efficiency, follows the theoretical rule in that a timine residue is located immediately upstream and a cystein downstream from the stop codon.

After confirming in vitro nonsense suppression in KS, our study goes one step further to investigate the in vivo effectiveness of gentamicin on two cultured patient LCLs carrying nonsense mutations. Consistent with our in vitro data, we showed that gentamicin treatment is able to partially restore a functional endogenous KMT2D protein confirming the use of the in vitro assay as a good model for evaluating drug-induced readthrough and selecting patient carrying nonsense mutations that could benefit from treatment. Nevertheless, studies on a larger number of KS cell lines are mandatory.

Our analysis indicated that some of *KMT2D*-truncating transcripts suffer NMD process contributing to instability of mutant mRNA and haploinsufficiency of KMT2D protein, thus it is possible that NMD minimizes the effect of readthrough treatment for some *KMT2D* transcripts. To overcome this undesirable contingent issue, the readthrough strategy might be combined with inhibition of NMD by specific inhibitors and/or siRNA directed against NMD key factors as UPF1 or UPF2.

Several other aminoglycosides and nonaminoglycosides agents are used for their nonsense suppression activity in various types of cell culture with a different efficiency. Our preliminary results encourage further testing with other compounds including additional aminoglycoside such as G418 and amikacin, and nonaminoglycosides agents with improved biocompatibility, such as PTC124 and RTC13, RTC14, and NB30 [Bordeira-Carrico et al., 2012].

## Conclusions

This study expanded the picture of *KMT2D* and *KDM6A* mutations that cause KS, adds some insight in the functional mechanisms that cause the disease, and finally provides the first preliminary proof-of-concept that naturally occurring nonsense mutations in *KMT2D* and *KDM6A* can be effectively suppressed providing a rational strategy for identifying patients likely to respond and therefore more likely to benefit from treatment with readthrough inducers.

## Supporting Information

Disclaimer: Supplementary materials have been peer-reviewed but not copyedited.

Figure S1. MLPA analysis of KMT2D gene. Figure S2. Schematic representation of KMT2D splicing mutations and their effect on cDNA patients. Figure S3. KMT2D mutations are regulated by NMD. Table S1. KMT2D and KDM6A mutations identified in our cohort of Kabuki patients. Table S2. In silico prediction of pathogenic effect of KMT2D# and KDM6A missense variants. Table S3. Values of the measured quantities and *p-values*. Table S4. Basal and gentamic-induced readthrough for KB mutations.
