# Supplementary material for: Molecular Analysis, Pathogenic Mechanisms, and Readthrough Therapy on a Large Cohort of Kabuki Syndrome Patients
Source: Hum Mutat. 2014 Mar 13;35(7):841–50. doi: 10.1002/humu.22547 (PMC4234006; doi:10.1002/humu.22547)
Supplement: Supplementary file 2 [file humu0035-0841-SD2.pdf]

A

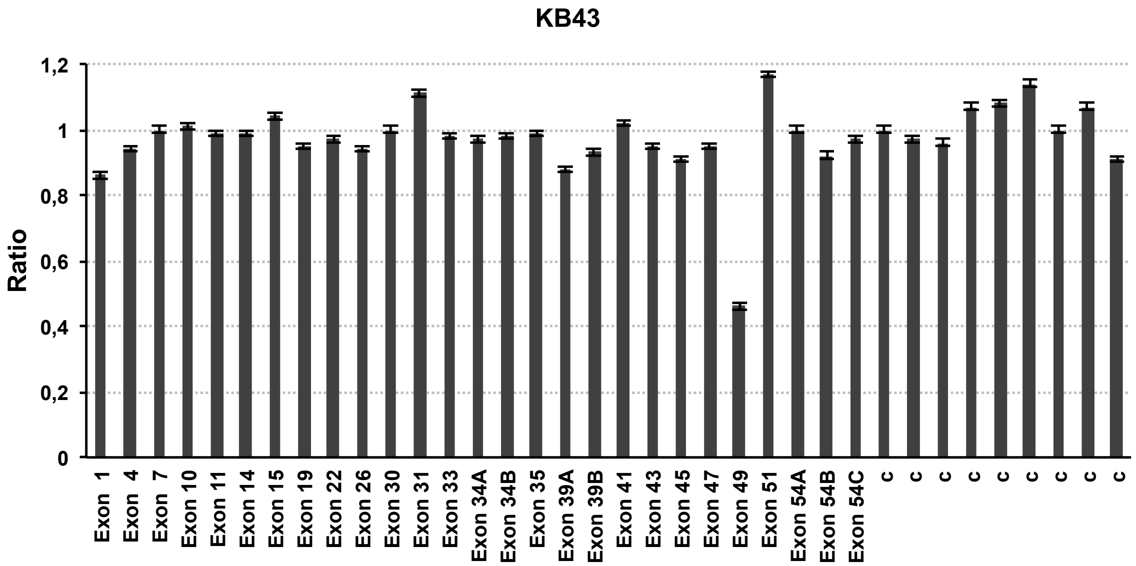

B

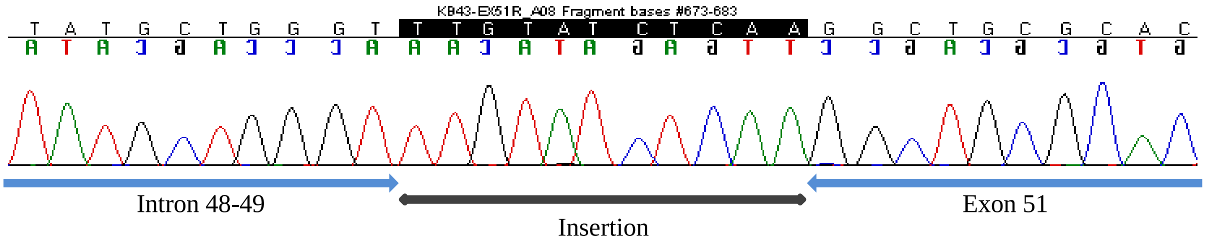

Supp. Figure S1. MLPA analysis of KMT2D gene. (A) Deletion of exon 49 was detected in the KB43 patient. (B) The electropherogram showing the boundaries of the deletion c.15785-238_16168del2425. An insertion of 10 nucleotides, TTGTATCTCAA, was identified between intron 48-49 and exon 51 (highlighted in black).

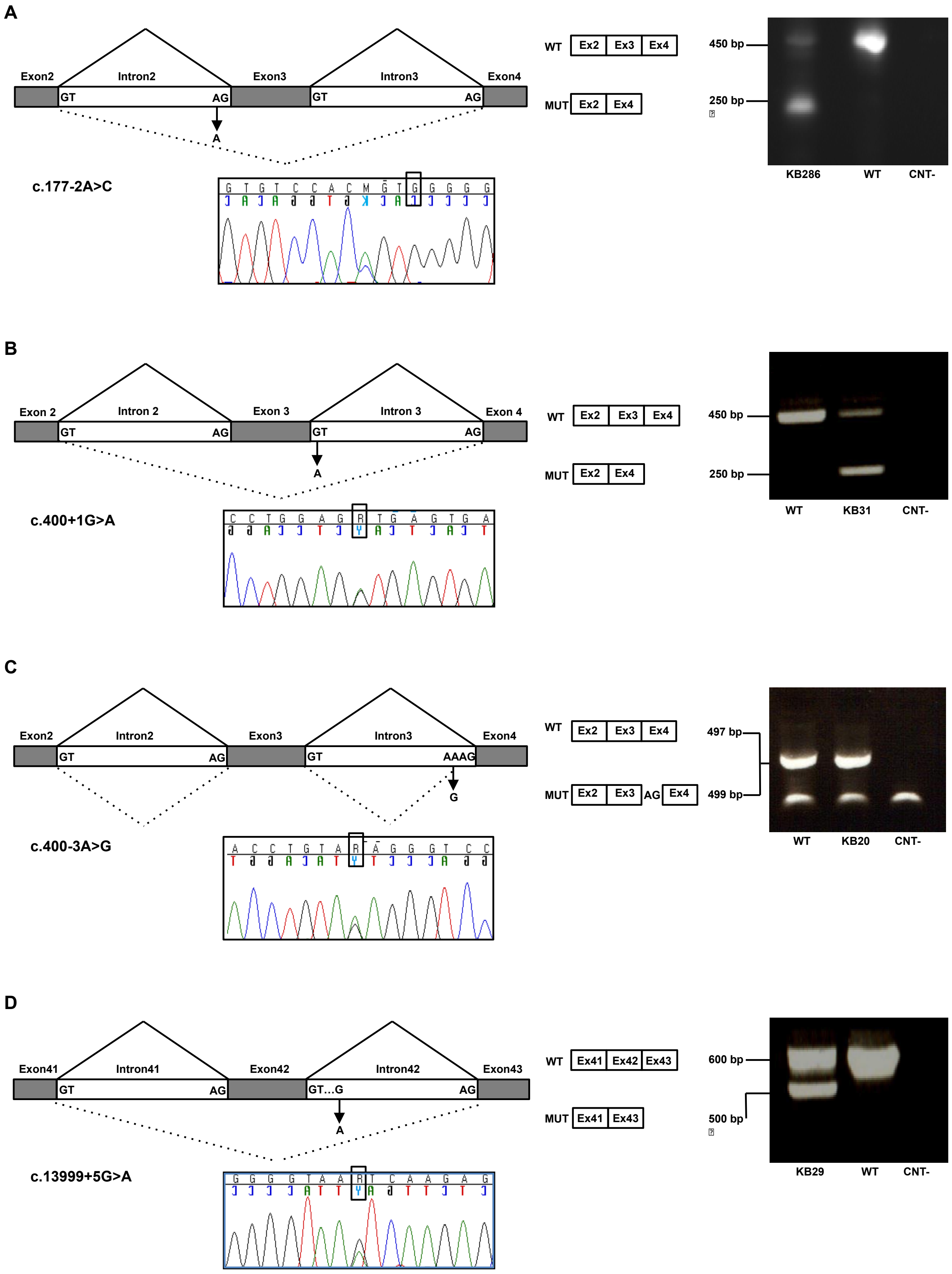

Supp. Figure S2

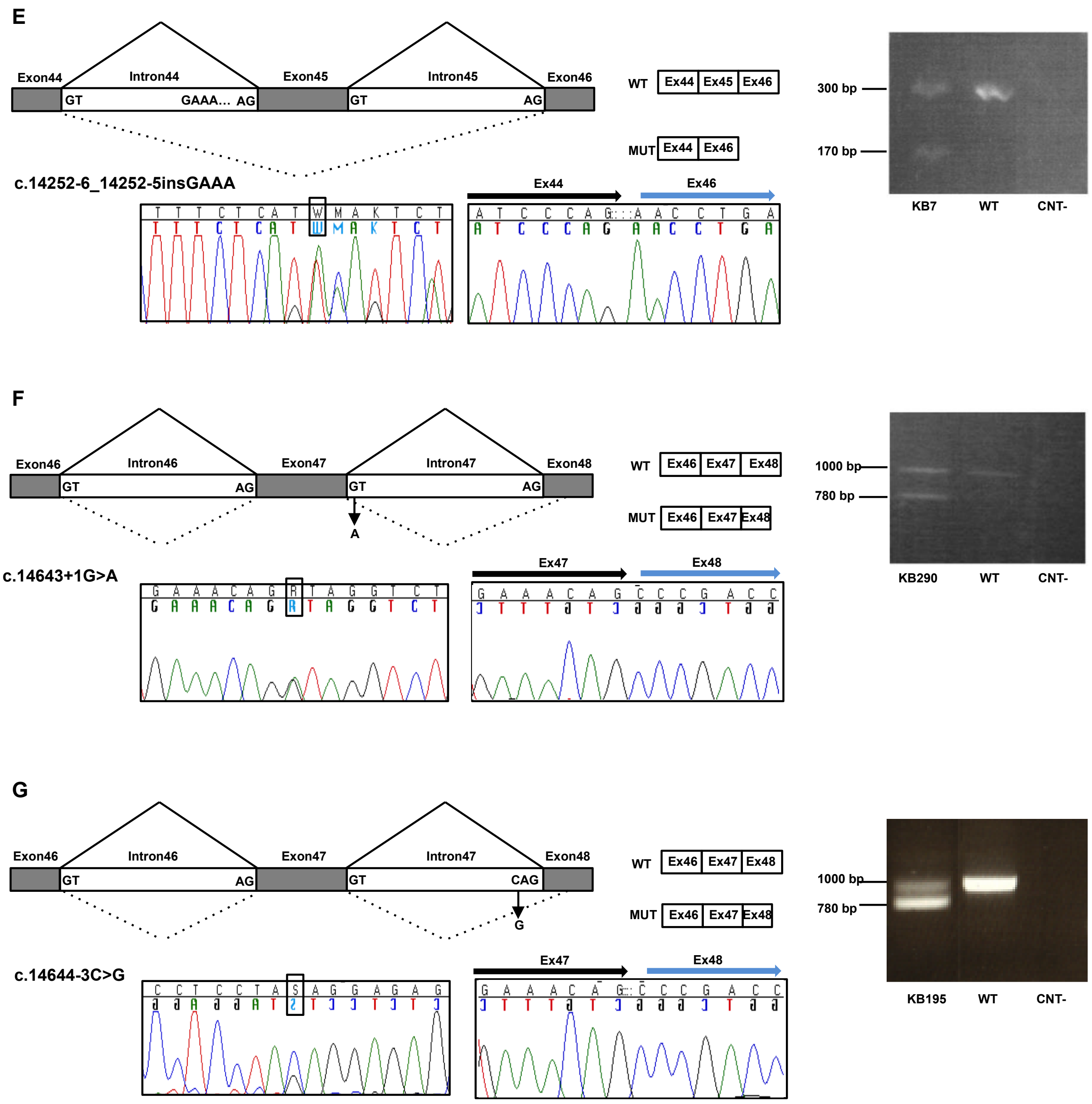

Supp. Figure S2. Schematic representation of KMT2D splicing mutations and their effect on cDNA patients. The electropherograms showing the KMT2D splicing heterozygous mutations in Kabuki patients are reported. RT-PCR analysis on cDNA from Kabuki patients and control (wt: wild type) are showed. Agarose gel images of PCR products showed both normal and aberrant spliced products. Patient cDNAs were used as templates for PCR amplifications in the regions displaying splicing mutations. See text for additional details.

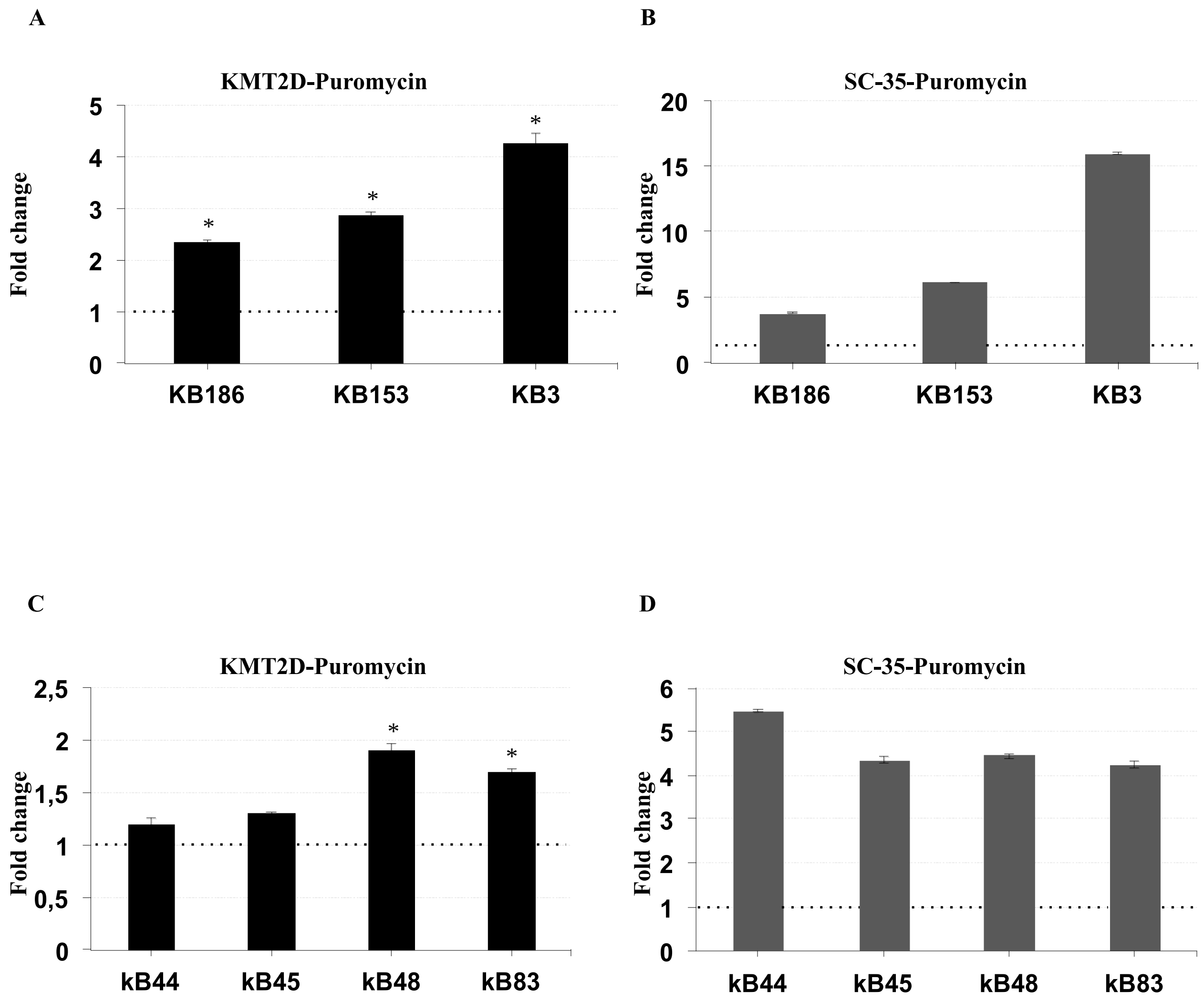

Supp. Figure S3

E

Normal control cDNA reverse

KB186 cDNA without puromycin reverse

KB186 cDNA with puromycin reverse

Normal control cDNA forward

KB186 cDNA without puromycin forward

KB186 cDNA with puromycin forward

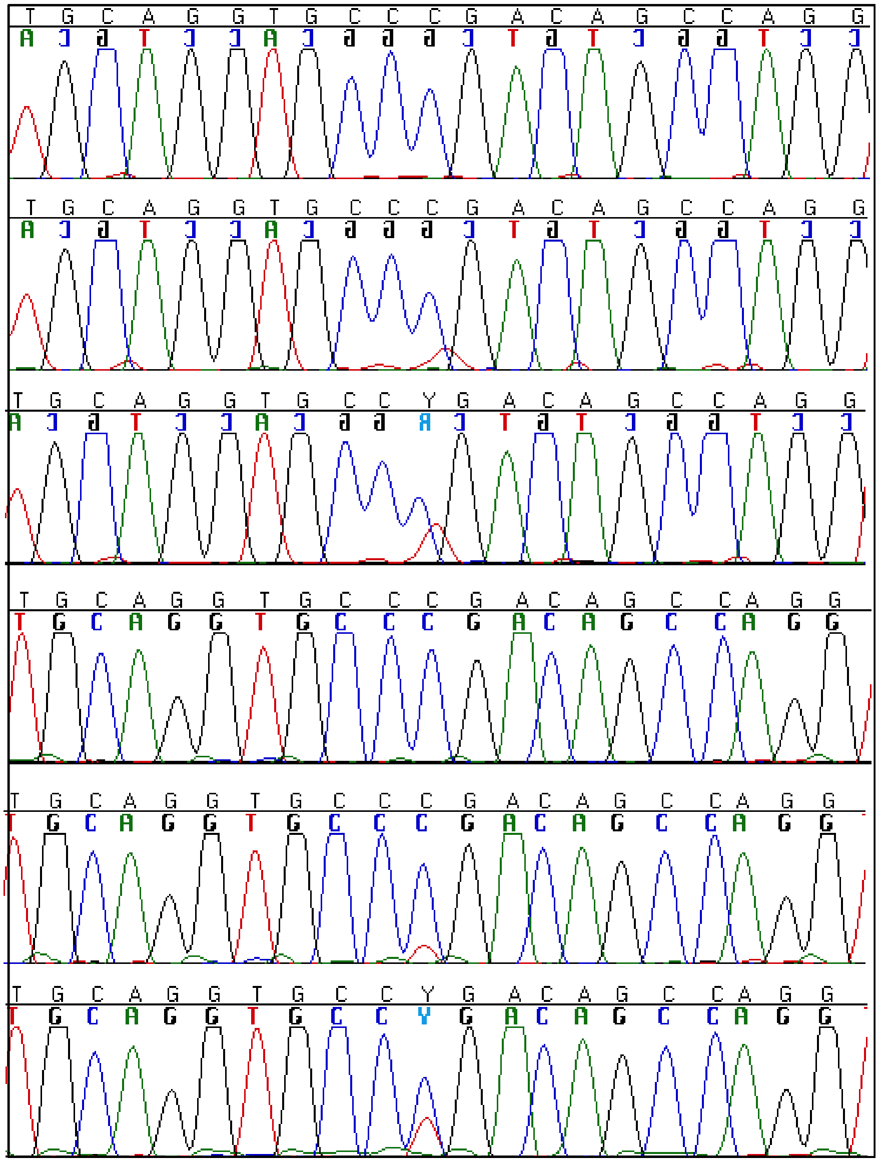

Supp. Figure S3

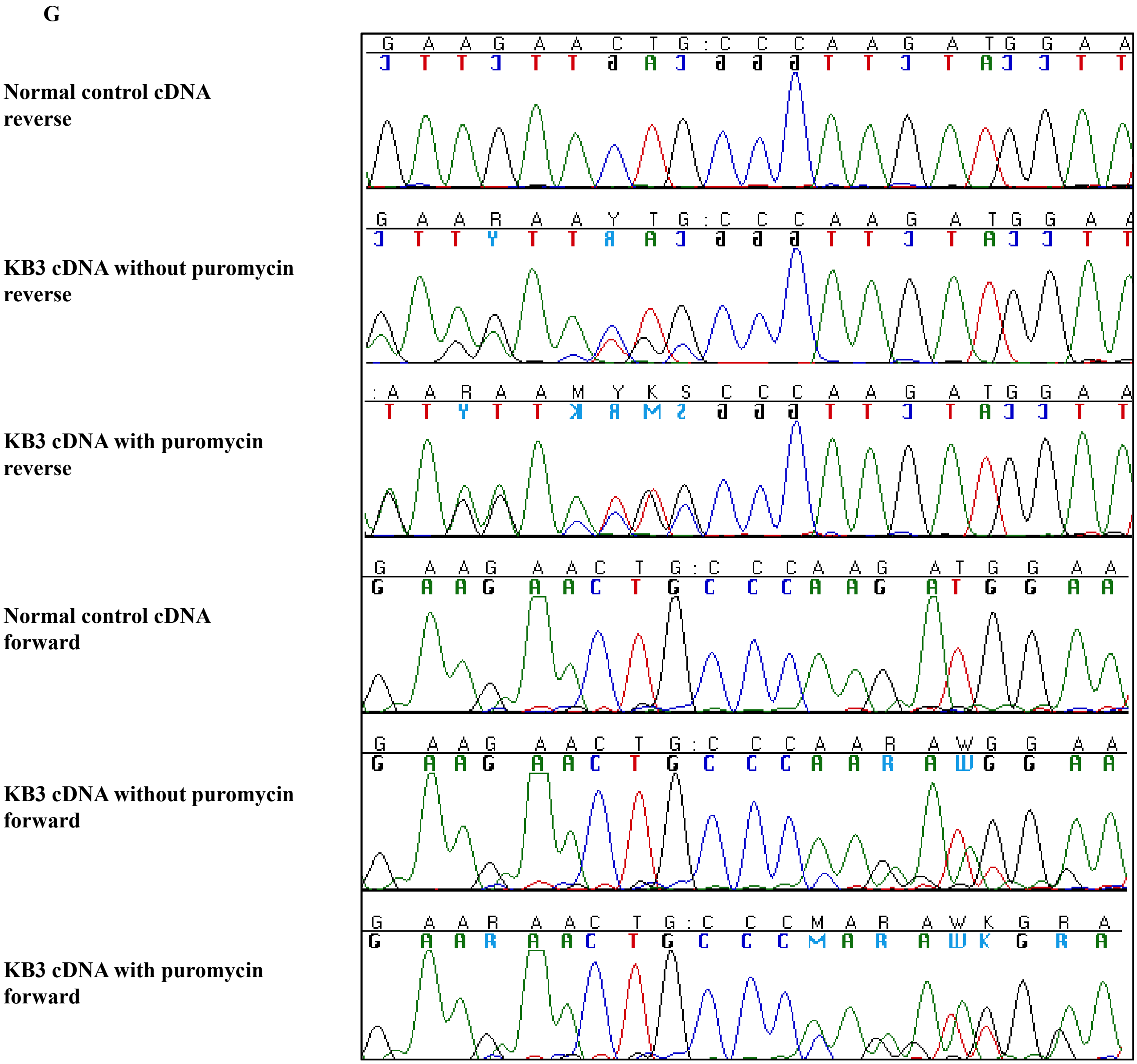

Supp. Figure S3. KMT2D mutations are regulated by NMD. **(A-D)** The mRNA levels of KMT2D in 3 KS-fibroblast and 4 KS-lymphoblastoid cell lines following treatment with puromycin (200ug/ml for 8 hours) were detected by using qPCR. The physiological NMD substrate SC-35 1.7 Kb was included as positive control.* P value<0.05. **(E-G)** In the presence of puromycin, the KMT2D mutant alleles (c.9961C>T, KB186 **(E)**, c.7903C>T, KB153 **(F)**; and c.5652dup, KB3 **(G)**) were restored. Without puromycin the mutant alleles were observed at a lower level than the wild type allele.

Supp. Table S1. KMT2D and KDM6A mutations identified in our cohort of Kabuki patients

Mutation Type	ID	Exon/ Intron	Mutation	AA change	Inheritance	Reference
**Patients with KMT2D mutations**						
**Nonsense**	KB49	5	c.669T>G	p.Tyr223X	NA	4, #
	KB35	10	c.1921G>T	p.Glu641X	NA	4, #
	KB33	16	c.4419G>A	p.Trp1473X	NA	4, #
	KB63	19	c.4895delC	p.Ser1632X	NA	4, #
	KB317	**22**	**c.5212G>T**	**p.Glu1738X**	**NA**	**This Study**
	KB262	**26**	**c.5674C>T**	**p.Gln1892X**	**NA**	**This Study**
	KB26	31	c.6295 C>T	p.Arg2099X	NA	1, 4, #
	KB66	31	c.7246C>T	p.Gln2416X	NA	4, #
	KB59	31	c.7903C>T	p.Arg2635X	de novo	4, #
	KB153, KB226	31	c.7903C>T	p.Arg2635X	de novo, de novo	4
	KB198	**31**	**c.7936G>T**	**p.Glu2646X**	**de novo**	**This Study**
	KB323	33	c.8311C>T	p.Arg2771X	NA	2,7
	KB289	34	c.8743C>T	p.Arg2915X	NA	3
	KB186	34	c.9961C>T	p.Arg3321X	de novo	1, 5, 7
	KB56	34	c.10135C>T	p.Gln3379X	de novo	4, #
	KB168	**39**	**c.10750C>T**	**p.Gln3584X**	**de novo**	**This Study**
	KB46	39	c.10841C>G	p.Ser3614X	de novo	4, #
	KB41, KB44	39	c.11119C>T	p.Arg3707X	NA, NA	4, #
	KB42	39	c.11269C>T	p.Gln3757X	de novo	4, #
	KB25	39	c.11434C>T	p.Gln3812X	NA	4, #
	KB244	39	c.11674C>T	p.Gln3892X	NA	7
	KB178	**39**	**c.11704C>T**	**p.Gln3902X**	**NA**	**This Study**
	KB181	**39**	**c.11869C>T**	**p.Gln3957X**	**NA**	**This Study**
	KB65	39	c.12076C>T	p.Gln4026X	NA	4, #
	KB40	39	c.12274C>T	p.Gln4092X	NA	4, #
	KB114	39	c.12274C>T	p.Gln4092X	de novo	4
	KB82	**39**	**c.12844C>T**	**p.Arg4282X**	**de novo**	**This Study**
	KB189	**39**	**c.12955A>T**	**p.Arg4319X**	**de novo**	**This Study**
	KB183	39	c.13450C>T	p.Arg4484X	de novo	2, 9
	KB175	**39**	**c.13507C>T**	**p.Gln4503X**	**de novo**	**This Study**
	KB73	40	c.13666A>T	p.Lys4556X	de novo	4, #
	KB83	**48**	**c.15022G>T**	**p.Glu5008X**	**NA**	**This Study**
	KB45, KB72	48	c.15079C>T	p.Arg5027X	NA, NA	2, 4, #
	KB130	52	c.16360C>T	p.Arg5454X	NA	1, 2, 5
**Frameshift**	KB75	4	c.472delT	p.Cys158ValfsX50	NA	4, #
	KB8	5	c.588delC	p.Cys197AlafsX11	NA	9
	KB58	6	c.705delA	p.Glu237SerfsX24	NA	4, #
	KB57	8	c.1035_1036delCT	p.Cys346SerfsX17	NA	4, #, 6*
	KB89	10	c.1345_1346delCT	p.Leu449ValfsX5	NA	4, #
	KB156	**10**	**c.1503dupT**	**p.Pro502SerfsX7**	**de novo**	**This Study**
	KB116	10	c.1634delT	p.Leu545ArgfsX385	NA	7
	KB48	11	c.2993dupC	p.Met999TyrfsX69	de novo	4, #
	KB203	**11**	**c.3161_3171delCGTTGAGTCCC**	**p.Pro1054HisfsX10**	**NA**	**This Study**
	KB309	**11**	**c.3730delG**	**p.Val1244SerfsX86**	**NA**	**This Study**
	KB142	**13**	**c.4021delG**	**p.Val1341LeufsX35**	**de novo**	**This Study**
	KB311	**14**	**c.4135_4136delAT**	**p.Met1379ValfsX52**	**NA**	**This Study**
	KB188	**16**	**c.4454delC**	**p.Pro1485LeufsX21**	**de novo**	**This Study**
	KB159	**19**	**c.4896_4905delAGATGCCCTT**	**p.Asp1633AlafsX86**	**NA**	**This Study**
	KB3	26	c.5652dupC	p.Lys1885GlnfsX18	NA	9, #
	KB84	26	c.5779delC	p.Gln1927LysfsX120	NA	4, #
	KB146	**27**	**c.5857delC**	**p.Leu1953TrpfsX94**	**de novo**	**This Study**
	KB208	**28**	**c.5954delC**	**p.Thr1985LysfsX62**	**de novo**	**This Study**
	KB221	**29**	**c.6149_6150delGA**	**p.Arg2050LysfsX6**	**de novo**	**This Study**
	KB152	**31**	**c.6583delA**	**p.Thr2195ProfsX69**	**de novo**	**This Study**
	KB267	31	c.6594delC	p.Tyr2199IlefsX65	NA	5
	KB79, KB102	31	c.6595delT	p.Tyr2199IlefsX65	de novo, de novo	1, 3, 4, 5, 7, #
	KB67	31	c.6638_6641delGCGC	p.Gly2213AlafsX50	de novo	4, #
	KB176	**31**	**c.6738delA**	**p.Lys2246AsnfsX18**	**NA**	**This Study**
	KB253	**31**	**c.6794delG**	**p.Gly2265GlufsX21**	**NA**	**This Study**
	KB278	**31**	**c.7481dupT**	**p.Ala2496SerfsX10**	**NA**	**This Study**
	KB313	**32**	**c.8196delG**	**p.Ser2733ValfsX24**	**NA**	**This Study**
	KB80	33	c.8273delG	p.Gly2758AlafsX29	NA	4, #
	KB243	**34**	**c.8430_8431insAA**	**p.Gln2811AsnfsX41**	**de novo**	**This Study**
	KB182	**34**	**c.9203delA**	**p.Gln3068GlyfsX3**	**NA**	**This Study**
	KB30	38	c.10606delC	p.Arg3536AlafsX122	NA	4, #
	KB101	39	c.11066_11078delCTGGATCCCTGGC	p.Ala3689ValfsX56	de novo	4, #
	KB172	**39**	**c.12647delC**	**p.Pro4216LeufsX62**	**NA**	**This Study**
	KB192	**39**	**c.12966delA**	**p.Gln4322HisfsX62**	**de novo**	**This Study**
	KB54	39	c.13129dupT	p.Trp4377LeufsX33	NA	4, #
	KB121	**39**	**c.13277dupT**	**p.Ala4428SerfsX59**	**de novo**	**This Study**
	KB123	**42**	**c.13884dupC**	**p.Thr4629HisfX18**	**de novo**	**This Study**
	KB197	**47**	**c.14592dupG**	**p.Pro4865AlafsX48**	**de novo**	**This Study**
	KB125	**48**	**c.15031delG**	**p.Glu5011SerfsX40**	**NA**	**This Study**
	KB 43[&]	**49-51**	**c.15785-238_16168del2425insTTGTATCTAA**		**NA**	**This Study**
	KB64	53	c.16469_16470delAA	p.Lys5490ArgfsX21	NA	4, #
	KB16	48	c.15374dupT ^	p.Met5124IlefsX14	de novo	9, #
**Indels**	KB274	**10**	**c.2532_2591del60**	**p.Arg845_Pro864del**	**NA**	**This Study**
	KB281	**39**	**c.11714_11716dupAGC**	**p.Gln3905dup**	**Maternal**	**This Study**
	KB71	39	c.11819_11836dup18	p.Leu3940_Gln3945dup	Maternal	4, #
	KB227	**39**	**c.11843_11860del18**	**p.Leu3948_Gln3953del**	**Paternal**	**This Study**
	KB228	**39**	**c.11854_11874dup21**	**p.Gln3952_Gln3958dup**	**Paternal**	**This Study**
	KB77	48	c.15163_15168dupGACCTG	p.Asp5055_Leu5056dup	NA	4, #
	KB53	53	c.16489_16491delATC	p.Ile5497del	NA	4, 7, #
**Missense**	KB21	**3**	**c.346T>C**	**p.Ser116Pro**	**Maternal**	**This Study**
	KB21	4	c.510G>C	p.Gln170His	Maternal	9#
	KB256	**5**	**c.626C>T**	**p.Thr209Ile**	**NA**	**This Study**
	KB269	10	c.1940C>A	p.Pro647Gln	Maternal	3
	KB215	**11**	**c.3392C>T**	**p.Pro1131Leu**	**Maternal**	**This Study**
	KB283	**11**	**c.3524C>T**	**p.Thr1175Ile**	**NA**	**This Study**

Mutation Type	ID	Exon/ Intron	Mutation	AA change	Inheritance	Reference
	KB222	**11**	**c.3572C>T**	**p.Pro1191Leu**	**Paternal**	**This Study**
	KB32	11	c.3773 G>A	p.Arg1258Gln	Paternal	4, #
	KB307	**14**	**c.4171G>A**	**p.Glu1391Lys**	**NA**	**This Study**
	KB28	15	c.[4249A>G; 4252C>A]	p.[Met1417Val; Leu1418Met]	Maternal	4, #
	KB174	**15**	**c.4283T>C**	**p.Ile1428Thr**	**Maternal**	**This Study**
	KB138	**16**	**c.4427C>G**	**p.Ser1476Cys**	**NA**	**This Study**
	KB34	16	c.4565 A>G	p.Gln1522Arg	Paternal	4, #
	KB177	**22**	**c.5226G>C**	**p.Glu1742Asp**	**NA**	**This Study**
	KB119, KB204	**31**	**c.6638 G>A**	**p.Gly2213Asp**	**Maternal, Maternal**	**This Study**
	KB326	**31**	**c.6811C>T**	**p.Pro2271Ser**	**NA**	**This Study**
	KB107	**31**	**c.6970C>A**	**p.Pro2324Thr**	**NA**	**This Study**
	KB122	31	c.7829T>C	p.Leu2610Pro	Maternal	6*
	KB27	34	c.8521 C>A	p.Pro2841Thr	NA	4, #
	KB326	**34**	**c.10192A>G**	**p.Met3398Val**	**NA**	**This Study**
	KB292	**37**	**c.10499G>T**	**p.Gly3500Val**	**de novo**	**This Study**
	KB86	**39**	**c.10966C>T**	**p.Arg3656Cys**	**NA**	**This Study**
	KB293	**39**	**c.11794C>G**	**p.Gln3932Glu**	**NA**	**This Study**
	KB204	**39**	**c.12070A>G**	**p.Lys4024Glu**	**Paternal**	**This Study**
	KB247	**39**	**c.12485G>A**	**p.Arg4162Gln**	**NA**	**This Study**
	KB107	**39**	**c.12488C>T**	**p.Pro4163Leu**	**NA**	**This Study**
	KB170	**39**	**c.13256C>T**	**p.Pro4419Leu**	**Maternal**	**This Study**
	KB86	**48**	**c.14893G>A**	**p.Ala4965Thr**	**NA**	**This Study**
	KB38	48	c.[15084C>G; 15100 T>G]	p.[Asp5028Glu; Phe5034Val]	de novo	4, #
	KB154, KB185	48	c.15088C>T	p.Arg5030Cys	de novo	9
	KB76	48	c.15176A>C	p.His5059Pro	de novo	4, #
	KB129	**48**	**c.15292A>C**	**p.Thr5098Pro**	**NA**	**This Study**
	KB171	48	c.15565G>A	p.Gly5189Arg	Maternal	11
	KB264	48	c.15640C>T	p.Arg5214Cys	NA	5, 7, 9
	KB24, KB219	48	c.15641G>A	p.Arg5214His	de novo, NA	1, 5
	KB109	**48**	**c.15649T>C**	**p.Trp5217Arg**	**de novo**	**This Study**
	KB17	50	c.16019 G>A	p.Arg5340Gln	de novo	4, #
	KB169	**51**	**c.16273G>A**	**p.Glu5425Lys**	**de novo**	**This Study**
	KB90	51	c.16295G>A	p.Arg5432Gln	NA	8
	KB177	**52**	**c.16412G>T**	**p.Arg5471Met**	**NA**	**This Study**
	KB120	**54**	**c.16528T>G**	**p.Tyr5510Asp**	**de novo**	**This Study**
**Splice-site**	KB286	**int 2-3**	**c.177-2A>C, r.177_400del224**	**p.Ser59ArgfsX86**	**de novo**	**This Study**
	KB31	int 3-4	c.400+1G>A, r.177_400del224	p.Ser59ArgfsX86	NA	4, #
	KB20	int 3-4	c.401-3A>G, r.400_401insAG	p.Gly134GlufsX75	de novo	4, #
	KB210	int 17-18	c.4693+1G>A, r.4681_4693del GTAAAGCCTGTGG	p.Val1561ArgfsX11	de novo	10, #
	KB29	int 42-43	c.13999+5G>A, r.13840_13999del160	p.Asn4614IlefsX5	NA	2, 4, 7, #
	KB7	int 44-45	c.14252-6_14252-5insGAAA, r.14252_14382del131	p.Val4751_GlufsX22	de novo	9, #
	KB290	**int 47-48**	**c.14643+1G>A, r.14644_14875del232**	**p.Glu4882ProfsX36**	**de novo**	**This Study**
	KB195	**int 47-48**	**c.14644-3C>G, r.14644_14875del232**	**p.Gln4882ProfsX36**	**de novo**	**This Study**
**Patients with KDM6A mutations**						
**Nonsense**	KB215	6	c.514C>T	p.Arg172X	de novo	12
**Frameshift**	KB39	**16**	**c.1846_1849delACTC**	**p.Thr616TyrfsX8**	**NA**	**This Study**
**Missense**	KB131	**20**	**c.2939A>T**	**p.Asp980Val**	**NA**	**This Study**
**Splice-Site**	KB127	**int 22-23**	**c.3284+3_3284+6delAAGT, r.3210_3284del75**	**p.Asn1070_Lys1094del**	**de novo**	**This Study**

Supp. Table S2. In silico prediction of pathogenic effect of KMT2D[#] and KDM6A missense variants

ID	Exon	Inheritance	AA change	POLYPHEN		UMD-predictor		ALIGN GVGD[§]	PROVEAN	SIFT
				prediction	score	prediction	score		cutoff=-2.5	cutoff=0.05
**KMT2D mutations**										
KB21	4	inherited (M)	p.Gln170His	Damaging	0.998	Polymorphism	35	Class C0	Neutral	Damaging
KB256	5	NA	p.Thr209Ile	Benign	0.096	Probably Polymorphism	59	Class C0	Neutral	Damaging
KB269	10	inherited (M)	p.Pro647Gln	Damaging	0.855	Probably Pathogenic	66	Class C0	Neutral	Tolerated
KB215	11	inherited (M)	p.Pro1131Leu	Benign	0.196	Probably Polymorphism	51	Class C0	Neutral	Damaging
KB283	11	NA	p.Thr1175Ile	Benign	0.022	Probably Polymorphism	59	Class C0	Neutral	Damaging
KB222	11	inherited (F)	p.Pro1191Leu	Damaging	0.764	Probably Polymorphism	51	Class C25	Deleterious	Damaging
KB32	11	inherited (F)	p.Arg1258Gln	Damaging	0.997	Polymorphism	44	Class C35	Neutral	Damaging
KB307	14	NA	p.Glu1391Lys	Damaging	1	Polymorphism	29	Class C65	Deleterious	Damaging
KB28	15	inherited (M)	p.Met1417Val	Damaging	0.476	Polymorphism	22	Class C15	Neutral	Tolerated
KB28	15	inherited (M)	p.Leu1418Met	Damaging	1	Polymorphism	15	Class C0	Neutral	Damaging
KB174	15	inherited (M)	p.Ile1428Thr	Damaging	0.889	Probably Pathogenic	73	Class C65	Neutral	Tolerated
KB138	16	NA	p.Ser1476Cys	Benign	0.005	Polymorphism	44	Class C65	Neutral	Tolerated
KB34	16	inherited (F)	p.Gln1522Arg	Damaging	0.997	Probably Polymorphism	59	Class C35	Deleterious	Damaging
KB177	22	NA	p.Glu1742Asp	Benign	0	Polymorphism	15	Class C0	Neutral	Tolerated
KB204	31	inherited (M)	p.Gly2213Asp	Damaging	0.454	Probably Pathogenic	66	Class C65	Neutral	Damaging
KB119	31	inherited (M)	p.Gly2213Asp	Damaging	0.454	Probably Pathogenic	66	Class C65	Neutral	Damaging
KB326	31	NA	p.Pro2271Ser	Benign	0.024	Probably Polymorphism	59	Class C65	Neutral	Damaging
KB107	31	NA	p.Pro2324Thr	Damaging	0.985	Probably Pathogenic	66	Class C35	Neutral	Damaging
KB122	31	inherited (M)	p.Leu2610Pro *	Benign	0.076	Probably Polymorphism	51	Class C65	Neutral	Tolerated
KB287	31	inherited (M)	p.Leu2610Pro *	Benign	0.076	Probably Polymorphism	51	Class C65	Neutral	Tolerated
KB27	34	NA	p.Pro2841Thr	Benign	0.009	Probably Pathogenic	66	Class C35	Neutral	Damaging
KB326	34	NA	p.Met3398Val	Benign	0	Polymorphism	29	Class C0	Deleterious	Damaging
KB292	37	de novo	p.Gly3500Val	Damaging	0.99	Pathogenic	88	Class C65	Deleterious	Damaging
KB86	39	NA	p.Arg3656Cys	Damaging	1	Pathogenic	81	Class C65	Neutral	Damaging
KB293	39	NA	p.Gln3932Glu	Benign	0.002	Polymorphism	15	Class C0	Neutral	Damaging
KB204	39	inherited (F)	p.Lys4024Glu	Benign	0.296	Polymorphism	29	Class C55	Neutral	Damaging
KB247	39	NA	p.Arg4162Gln	Benign	0.003	Polymorphism	48	Class C0	Neutral	Damaging
KB107	39	NA	p.Pro4163Leu	Damaging	0.991	Probably Polymorphism	51	Class C65	Neutral	Damaging
KB170	39	inherited (M)	p.Pro4419Leu	Damaging	1	Probably Polymorphism	51	Class C65	Deleterious	Damaging
KB86	48	NA	p.Ala4965Thr	Damaging	0.941	Probably Polymorphism	59	Class C55	Neutral	Tolerated
KB38	48	de novo	p.Asp5028Glu	Damaging	0.999	Polymorphism	22	Class C35	Deleterious	Damaging
KB154	48	de novo	p.Arg5030Cys	Damaging	1	Pathogenic	81	Class C65	Deleterious	Damaging
KB185	48	de novo	p.Arg5030Cys	Damaging	1	Pathogenic	81	Class C65	Deleterious	Damaging
KB38	48	NA	p.Phe5034Val	Damaging	0.999	Polymorphism	44	Class C45	Deleterious	Damaging
KB76	48	de novo	p.His5059Pro	Damaging	1	Probably Pathogenic	73	Class C65	Deleterious	Damaging
KB129	48	NA	p.Thr5098Pro	Damaging	0.992	Probably Pathogenic	66	Class C35	Deleterious	Tolerated
KB171	48	inherited (M)	p.Gly5189Arg	Damaging	1	Pathogenic	88	Class C65	Deleterious	Damaging
KB264	48	NA	p.Arg5214Cys	Damaging	1	Pathogenic	81	Class C65	Deleterious	Damaging
KB219	48	NA	p.Arg5214His	Damaging	1	Polymorphism	37	Class C25	Deleterious	Damaging
KB24	48	de novo	p.Arg5214His	Damaging	1	Polymorphism	37	Class C25	Deleterious	Damaging
KB109	48	de novo	p.Trp5217Arg	Damaging	1	Probably Pathogenic	73	Class C65	Deleterious	Damaging
KB17	50	de novo	p.Arg5340Gln	Damaging	1	Polymorphism	37	Class C35	Deleterious	Damaging
KB169	51	de novo	p.Glu5425Lys	Damaging	1	Polymorphism	29	Class C55	Deleterious	Damaging
KB90	51	NA	p.Arg5432Gln	Damaging	1	Polymorphism	44	Class C35	Deleterious	Damaging
KB177	52	NA	p.Arg5471Met	Damaging	1	Pathogenic	100	Class C0	Deleterious	Damaging
KB120	54	de novo	p.Tyr5510Asp	Damaging	1	Pathogenic	81	Class C0	Deleterious	Damaging
**KDM6A mutations**										
KB131	20	de novo	p.Asp980Val	Damaging	1	NA	NA	Class C65	Deleterious	Damaging

* Kabuki-unrelated mutation, found in diffuse large B-cell lymphoma.
# RefSeq NM_003482.3
§ the score ranges from C0 (least likely interfere with function) to C65 (most likely interfere with function)

Supp. Table S3. Values of the measured quantities and *p-values*

A

Sample	KMT2D
**KB44**	0,455* (p=0,0003)
**KB45**	0,452* (p=0,0003)
**KB83**	0,386* (p=0,0002)
**KB41**	0,657 (p=0,0020)
**KB48**	0,352 (p=0,0001)
**KB82**	0,634 (p=0,0020)
**KB186**	0,702* (p=0,0020)
**KB153**	0,579* (p=0,0013)
**KB3**	0,660* (p=0,0015)

B

Sample	HOXC6	S100A2	S100A4	S100A5	S100A6
**KB41**	0,144* (p=0,0001)	0,954 (p=0,83620)	0,896 (p=0,0409)	0,798 (p=0,0043)	1,037 (p=0,0271)
**KB44**	0,427* (p=0,0003)	0,608* (p=0,0011)	0,423* (p=0,0000)	0,665* (p=0,0018)	0,241* (p=0,0000)
**KB45**	0,034* (p=0,0000)	0,259* (p=0,0001)	0,847* (p=0,0054)	0,662* (p=0,0026)	0,587* (p=0,0000)
**KB48**	0,560* (p=0,0007)	0,545* (p=0,0012)	0,634* (p=0,0002)	0,381* (p=0,0002)	0,296* (p=0,0000)
**KB82**	0,052* (p=0,0010)	0,563* (p=0,0008)	0,574* (p=0,0000)	1,258 (p=0,1348)	0,889 (p=0,0129)
**KB290**	0,297* (p=0,0003)	0,844 (p=0,0537)	0,582* (p=0,0003)	0,708* (p=0,0062)	0,683* (p=0,0000)

C

Sample	HOXC6
**KB153**	0,167* (p=0,0003)
**KB186**	0,859 (p=0,012)
**KB3**	0,609* (p=0,0009)
**KB153+E2**	0,324* (p=0,0000)
**KB186+E2**	0,607* (p=0,0003)
**KB3+E2**	0,776* (p=0,009)

D

Sample	Luciferase activity
**KB153**	0,293* (p=0,0003)
**KB186**	0,904 (p=0,0201)
**KB3**	0,579* (p=0,0021)
**KB153+E2**	0,156* (p=0,0003)
**KB186+E2**	0,519* (p=0,0002)
**KB3+E2**	0,375* (p=0,0013)

E

Sample	KMT2D
**KB153**	2,935* (p=0,0000)
**KB186**	2,186* (p=0,0000)
**KB3**	4,010* (p=0,0006)

F

Sample	KMT2D
**KB44**	1,192 (p=0,011)
**KB45**	1,345 (p=0,011)
**KB48**	1,932* (p=0,0000)
**KB83**	1,691* (p=0,0001)

(A) Protein level of KMT2D in 6 KS-LCL and 3 KS-fibroblast cell lines as showed in Figure 1A-F; **(B)** *HOXC6, S100A2, S100A4, S100A5, S100A6* expression level in 6 KS-LCL cells reported in Figure 1G; **(C)** *HOXC6* transcriptional level in 3 KS-fibroblast cells treated or not with E2 (Figure 2A-B); **(D)** Luciferase activity in 3 KS-fibroblast cells transfected with pGL3-ERE1-ERE2 luciferase-based reporter vector containing the *HOXC6* EREs sequences, treated or not with E2 reported in Figure 2C-E; **(E-F)** mRNA levels of *KMT2D* in 3 KS-fibroblast (E) and 4 KS-LCL lines (F) following treatment with puromycin (Supp. Figure S3A and C). *p-values* <0.01 are marked by asterisk.

Supp. Table S4. Basal and gentamic-induced readtrough for KB mutations

Gene	WT codon	Mutation	Nt change	Nucleotidic context of stop mutation	Readthrough at gentamicin (µg/ml)=%				Increase factor between doses 0 and 600	Increase factor between doses 0 and 800	Increase factor between doses 0 and 1200
					0	600	800	1200			
KMT2D	TAT	p.Tyr223X	c.669T>G	TGGGAGGGGGCTGCA**TAG**CTGGAGGAGGCTCCA	0,002	0,009*	0,004	0,004	4	2	2
KMT2D	TGG	p.Trp1473X	c.4419G>A	TGGTGGAAGTGCAAG**TGA**TGTGTGTCCTGT CCA	0,187	0,104	0,089	0,096	1	0	1
KMT2D	CGA	p.Arg2099X	c.6295 C>T	TGGCGTAAGACTGAC**TGA**CCGGCCCTACATCCA	0,022	0,398*	0,436*	0,356*	18	19	16
KMT2D	CAG	p.Gln2416X	c.7246C>T	TGGCCTGCCAGTCCT**TAG**TCCCAGTCCAGCCCA	0,013	0,037*	0,047*	0,062*	3	4	5
KMT2D	CGA	p.Arg2635X	c.7903C>T	TGGGGAGCCTCACAG**TGA**TCAGGCATCACC CCA	0,015	0,147*	0,135*	0,267*	10	9	18
KMT2D	GAA	p.Glu2646X	c.7936C>T	TGGGTCGAAAAGCGA**TAA**GACCCAGGGACTCCA	0,008	0,019*	0,026*	0,026*	3	3	3
KMT2D	CGA	p.Arg3321X	c.9961C>T	TGGCTTGCAGGTGCC**TGA**CAGCCAGGTTTGCCA	0,082	0,681*	0,887*	0,985*	8	11	12
KMT2D	CAG	p.Gln3379X	c.10135C>T	TGGCCAGTGCTATCA**TAG**AAGCCCATGGGCCCA	0,003	0,008*	0,011*	0,013*	2	3	3
KMT2D	CAG	p.Gln3584X	c.10750C>T	TGGCAGGTCCGGAAA**TAG**CAGAAGGAGCACCCA	0,049	0,213*	0,241*	0,269*	4	5	6
KMT2D	CGA	p.Arg3707X	c.11119C>T	TGGAACCTTGCTCTT**TGA**AGCCTCGGACCTCCA	0,014	0,079*	0,099*	0,098*	6	7	7
KMT2D	CAG	p.Gln3757X	c.11269C>T	TGGATCCAGCAGCAA**TAG**CAGCAGGGTCCTCCA	0,015	0,141*	0,117*	0,249*	10	8	17
KMT2D	CAG	p.Gln4092X	c.12274C>T	TGGTCTCTGCAGCTG**TAG**CCACCTCTGAGGCCA	0,080	0,135	0,145	0,152	2	2	2
KMT2D	CGA	p.Arg4282X	c.12844C>T	TGGGGGGCAGGGCCT**TGA**CCTCAGGGCCCACCA	0,036	0,545*	0,652*	0,967*	15	18	27
KMT2D	CGA	p.Arg5027X	c.15079C>T	TGGGACAAGGTACCG**TGA**GACATGCGTCGCCCA	0,090	0,119	0,103	0,168	1	1	2
KDM6A	CGA	p.Arg519X	c.1555C>T	TGGGTTGCACAGGTA**TGA**TCTACTGGAATTCCA	0,018	0,069*	0,061*	0,072*	4	3	4
KDM6A	TGG	p.Trp1239X	c.3717G>A	TGGAACAACATTGCT**TGA**AATGTTGGTCCACCA	0,006	0,000	0,001	0,001	0	0	0
	TQ: in frame Ctrl			GCAGGAACACAACAGCAATTACAG	18,122	14,865	15,635	12,372	1	1	1
	CGA^	p.Ser319X	c.956C>G	AGCCCATTTCCTTGACAGCATTTGGAA	0,027	0,299*	0,344*	0,454*	11	13	17

The readtrough response was defined as the factor of increase between basal and gentamicin-induced reatrough levels.

TQ: construct with no stop codon (100% of activity)
^: Duchenne muscolar dystrophy gene mutation used as positive control
°: Miyake, et al., 2013b
*: *p-value* <0.01
